# Pacemaker Implantation in a Patient with Isolated Persistent Left Superior Vena Cava Draining into the Left Atrium: A Case Report and Brief Literature Review

**DOI:** 10.3390/diagnostics12112707

**Published:** 2022-11-05

**Authors:** Iuliana-Claudia Totorean, Cristina Vacarescu, Dragoș Cozma, Constantin-Tudor Luca, Horea Feier, Mihai-Andrei Lazăr, Maria-Anastasia Deme, Svetlana Stoica, Diana-Aurora Arnautu, Dan Gaiță

**Affiliations:** 1 Cardiology Department, “Victor Babes” University of Medicine and Pharmacy, 2 Eftimie Murgu Sq., 300041 Timisoara, Romania; 2 Institute of Cardiovascular Diseases Timisoara, 13A Gheorghe Adam Street, 300310 Timisoara, Romania; 3 Research Center of the Institute of Cardiovascular Diseases Timisoara, 13A Gheorghe Adam Street, 300310 Timisoara, Romania

**Keywords:** persistent left superior vena cava, abnormal drainage, left atrium, pacemaker implantation, epicardial lead

## Abstract

Anomalies of the thoracic venous system are rare and usually discovered incidentally, but they become clinically relevant in the case of patients requiring cardiac device implantation. Persistent left superior vena cava is considered the most common venous drainage abnormality, with several anatomical variants that generate technical difficulties during pacemaker or defibrillator lead placement. We report a case of an isolated persistent left superior vena cava with abnormal drainage into the left atrium, associated with a hypoplastic right-sided superior vena cava, in a patient scheduled for permanent pacemaker implantation. Considering the patient’s anatomical characteristics, a transvenous approach proved unfeasible and the procedure was successfully accomplished via the surgical placement of a left ventricle epicardial lead. We aim to emphasize the clinical importance of such venous anomalies and to discuss the practical implications and challenges derived from these types of conditions, especially in the field of electrophysiology.

## 1. Introduction

Thoracic systemic venous anomalies represent a heterogeneous group of vascular abnormalities that are usually asymptomatic. They are usually asymptomatic if not associated with other congenital heart diseases, and they are discovered incidentally during vascular interventions or surgical procedures.

Persistent left superior vena cava (PLSVC) is the most common venous drainage anomaly of the systemic thoracic veins, with its prevalence among the general population ranging from 0.5 to 2%. In 80–90% of the cases, the PLSVC drains into the right atrium via a dilated coronary sinus [1,2,3,4].

PLSVC is commonly associated with a normal right-sided superior vena cava. However, in 10–20% of cases, an isolated PLSVC may be present, frequently coexisting with other cardiac congenital disorders or arrhythmias [4,5,6,7].

In up to 10% of the patients with PLSVC, it is possible for the vein to drain into the left atrium via an unroofed coronary sinus or directly into the left atrium, an even more uncommon condition [7,8]. A right-to-left shunt is thus formed, and the patient is susceptible to several complications including cyanosis, cerebrovascular embolism (especially venous air embolism after injections into the left arm) and heart failure [7,9,10,11,12,13].

We report the case of a patient with recurrent syncope and a significant cardiac conduction disorder, scheduled for conventional pacemaker implantation. This procedure proved unfeasible due to a persistent left superior vena cava draining aberrantly into the left atrium and a hypoplastic right-sided superior vena cava. These circumstances eventually required surgery for epicardial lead placement. To the best our knowledge, this is the first case reported in the literature that associates this type of venous anomaly and pacemaker implantation using a surgical approach. Additionally, we conducted a brief literature review on the current state of research regarding this topic, highlighting the need for the cardiologists to be knowledgeable about the broad spectrum of vascular abnormalities that may be found in patients requiring cardiac devices.

## 2. Case Report

A 65-year-old man, without any significative medical history, was admitted to our cardiology department due to two episodes of syncope one week prior to admission. A physical examination revealed resting hypoxia (oxygen saturation of 93% on room air), with no other relevant clinical signs. The patient had no family history of congenital heart disease. Electrocardiography showed a sinus rhythm, a QRS duration of 160 ms, complete right bundle branch block, left anterior fascicular block and first-degree atrioventricular block with a PR interval of 240 ms. A transthoracic echocardiography showed normal LV systolic function with an ejection fraction of 55%, mild valvular abnormalities, a slightly enlarged left atrium and no other additional findings. A 24 h ECG Holter monitoring revealed an intermittent third-degree atrioventricular block. Considering the correlation between the symptoms and the electrocardiographic findings, the patient was scheduled for conventional permanent pacemaker implantation.

**Figure 1:**
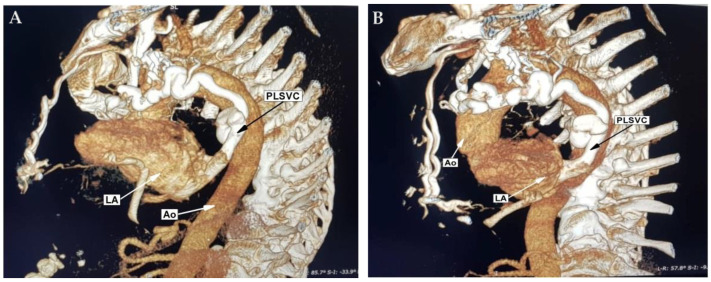
(**A**,**B**) Contrast-enhanced computed tomography—3D reconstructed images showing the dilated tortuous PLSVC draining into the left atrium. Abbreviations: LA, left atrium; Ao, aorta; PLSVC, persistent left superior vena cava.

Left subclavian access was initially used and several attempts to advance the guide wire into the left brachiocephalic vein and superior vena cava were made, without success. Therefore, a venography was performed, revealing a possible persistent left superior vena cava, but the drainage into the right atrium via the coronary sinus could not be visualized. Moreover, no contrast inflow into the right superior vena cava was demonstrated. A second attempt to place the pacemaker lead was made, using right subclavian vein access. A venography revealed the absence of a normal superior vena cava on the right side and the presence of tortuous intrathoracic collaterals, without any favorable anatomic variant for pacemaker lead placement. A complex thoracic venous anomaly was suspected, and the patient was referred to the radiology department for contrast-enhanced computed tomography reconstruction of the thoracic venous system. CT reconstruction revealed a hypoplastic superior vena cava with 2–3 mm in diameter, right-sided collaterals with significant tortuosity and abnormal drainage of the left brachiocephalic vein into the left inferior pulmonary vein and subsequently into the left atrium, via a dilated tortuous persistent left superior vena cava (Figure 1).

**Figure 2:**
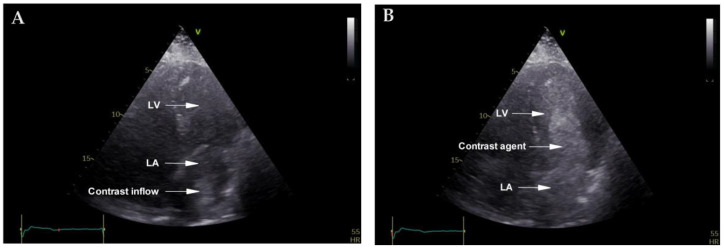
(**A**) Contrast transthoracic echocardiography, with an apical 4-chamber view showing contrast agent entering the left atrium via PLSVC drainage into the left inferior pulmonary vein. (**B**) Contrast transthoracic echocardiography, with an apical 4-chamber view showing early opacification of left atrium and left ventricle. Abbreviations: LA, left atrium; LV, left ventricle. N.B. The contrast agent was injected into the left arm.

Before the CT result, a transthoracic echocardiography (TTE) with agitated saline injected into the left cubital vein was also performed, revealing the contrast substance initially draining into the left inferior pulmonary vein and early opacification of the left atrium, thus confirming the right-to-left shunt. Due to the complex venous anomaly suspected, we have taken extra safety measures when the contrast substance was injected (Figure 2).

**Figure 3:**
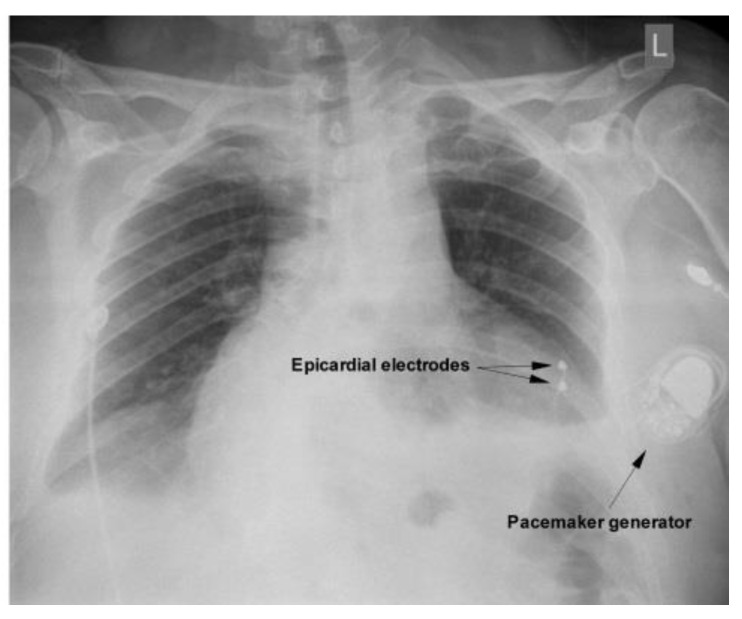
Follow-up chest radiography after pacemaker implantation.

The CT imaging confirmed that transvenous pacemaker implantation was not feasible for the patient; therefore, a surgical approach for epicardial lead placement was considered. The patient underwent general anesthesia, and a left lateral thoracotomy was performed. A ventricular lead with two epicardial electrodes was fixed on the diaphragmatic left ventricular surface; the lead was tunneled with care and connected to the generator, which was secured in a pocket anterior to the pectoralis major muscle. Postprocedural implant measurements showed normal lead impedance, as well as excellent pacing and sensing thresholds (0.5 V/15 mV). A chest radiography performed after the procedure showed the normal position of the pacemaker (Figure 3). The patient had a good recovery and was discharged one week after surgery.

Follow-up medical reevaluation was performed at one and six months after the procedure. The patient proved to be in good clinical condition, without the recurrence of symptoms, the pacemaker interrogation revealed adequate ventricular pacing and the echocardiography was stationary.

Reporting this case was conducted in accordance with the Declaration of Helsinki and approved by the Ethics Committee of the Timisoara Institute of Cardiovascular Diseases.

## 3. Discussion

In this case report, we aimed to present our approach for pacemaker implantation in a patient without adequate vascular access for transvenous lead placement due to a complex venous anomaly of the thoracic venous system, requiring a surgical strategy. Although we have experience with cardiac device implantation in patients with persistent left superior vena cava with normal venous drainage into the right atrium, this was the first case with such significant venous return abnormality diagnosed and managed in our center. Therefore, we aimed not only to present the case, but also to conduct a brief review in the following paragraphs of the main findings and challenges reported in the literature related to the topic.

Pacemaker implantation in patients with anomalies of the cardiac venous drainage is technically challenging since most of these anomalies are usually asymptomatic and discovered incidentally during the intervention. The potentially problematic spectrum is broad and can range from simple, isolated abnormalities to more complex ones [1], associated with other congenital heart anomalies [2]. Acquired anomalies of the thoracic venous system can also be technically challenging in the area of electrophisiology and interventional cardiology. Bismark et al. described a case of SVC occlusion in a patient with radiotherapy for thimic carcinoma who required implantation of a temporary pacemaker after a transcatheter aortic valve replacement procedure [3]. A history of cancer in the presence of other clinically relevant signs should always raise concerns about vena cava syndrome.

The most frequent abnormality related to the venous drainage system is PLSVC which can be found in 0.5–2% of the general population. There are several anatomic variants of PLSVC reported in the literature, as follows: double superior vena cava without any anastomosis, double superior vena cava with a transverse anastomosis between two venae cavae and left superior vena cava with absent right superior vena cava. The latter condition is referred to as isolated PLSVC, a significantly rare congenital disorder [1,2,3,4,5,6,14,15,16,17].

In 46% of cases, PLSVC (especially the isolated type) is associated with other congenital cardiac defects [4]. Lendzian et al. reported that anomalies of the caval veins are more often related to complex congenital heart disease, including: atrioventricular septal defects, tetralogy of Fallot, double outlet left ventricle, double outlet right ventricle, hypoplastic left heart, hypoplastic right heart, pulmonary atresia, single atrium, single ventricle or total anomalous pulmonary venous return [5].

Generally (80–90%), the left superior vena cava drains into the right atrium via a dilated coronary sinus and is an incidental finding, without hemodynamic consequences. However, such an anomaly becomes clinically relevant in circumstances that require pacemaker or defibrillator implantation, central venous access or cardiopulmonary bypass [1,2,3,4].

Transthoracic echocardiography has an important role in diagnosing a PLSVC, because a dilated coronary sinus observed during a routine TTE should always raise the suspicion of such a venous anomaly. Contrast TTE and transesophageal echocardiography can provide additional information [17,18]. Venography is another essential tool used in these patients, particularly if pacemaker or defibrillator implantation is necessary, since it helps diagnose the anomaly and also guides the procedure. When suspecting a more complex venous anomaly, contrast-enhanced computed tomography or magnetic resonance angiography are recommended for a more comprehensive evaluation [18,19].

Anomalies of cardiac venous drainage have been reported over the years in association with abnormalities of the cardiac conduction system in many studies, particularly PLSVC in patients with conduction disorders (AV blocks, junctional rhythm). Moreover, Wolff Parkinson White syndrome, AV nodal re-entry tachycardia or AV tachycardia have been correlated with left superior vena cava draining into the coronary sinus through an abnormal connection. Researchers postulate that this connection can be traced back to early intrauterine development period. The pacemaking tissue of the heart originates from two sites situated near the progenitors of the superior vena cava; the sinoatrial node is formed from the right-sided site, and the left-sided site is normally carried down to an area near the coronary sinus. Conduction system abnormalities may be related to coronary sinus anomalies, due to the adjacency of the coronary sinus to the left-sided primitive pacemaking tissue. Thus, the presence of a left-sided superior vena cava that leads to dilation of the coronary sinus could explain the association between this venous anomaly and abnormalities in the cardiac conduction tissue [20,21,22].

When a permanent pacemaker or defibrillator implant is needed, lead placement via a PLSVC may be challenging and skill-demanding, especially for unexperienced operators. However, these procedures are usually accomplished with favorable outcomes regarding device performance. As far as vein access, the left subclavian vein is recommended because lead manipulation is easier. From the technical point of view, since there is an acute angle between the coronary sinus ostium and the tricuspid valve, the lead should be looped in the right atrium and then advanced into the right ventricle. Active fixation leads and hand-shaped stylets are recommended in order to overcome technical difficulties [23,24,25,26]. Many cases of single chamber pacemaker implantation via a PLSVC have been reported over the years, but also there have been published cases where dual chamber pacemaker and defibrillator implantation was achieved via PLSVC, and a few reports of successful cardiac resynchronization therapy [27,28,29,30,31]. Parikh et al. reported an interesting case of CRT-D in a patient with PLSVC associated with an oclusive Thebesian valve, emphasizing the need for a better knowledge of cardiac embryology, anatomy and physiology in interventional cardiology [32]. Nevertheless, it is important to mention the iliac vein as a rescue solution for pacing in selected patients with limited central venous access [33].

Searching furthermore into the literature, reports showed that in up to 10% of patients with PLSVC, it is possible that the vein drains into the left atrium via an unroofed coronary sinus or directly into the left atrium, an even more uncommon condition. Consequently, a right-to-left shunt is created, and the patient is prone to develop several complications, including cyanosis, heart failure and venous air embolism, following injections into the left arm [7,8,9,10,11,12,13,14,15]. During the embryological period, if developmental arrest occurs at an earlier stage, the coronary sinus may be absent, leading to PLSVC drainage into the left atrium; although very rare, there has been evidence of PLSVC drainage into the left atrium in the presence of a normal coronary sinus [7,8,9,10,11,12,13,14,15].

There are a few isolated cases published so far regarding aberrant PLVSC drainage into the left atrium, but we did not identify another case reported so far that combines hypoplastic right-sided superior vena cava with PLSVC drainage into the left atrium and the need for pacemaker implantation. Yousaf et al. and Benz et al. each reported a case of persistent left superior vena cava draining into the left atrium, but with an otherwise normal course of the right superior vena cava and a normal heart [4,34]. Hulten et al. presented two cases of multimodality imaging of a right superior vena cava connecting to the left atrium and of an inferior vena cava connecting to the left atrium, both undergoing surgical correction [35]. Zhang and collaborators reported a case of total anomalous systemic venous drainage in which the right superior vena cava, persistent left superior vena cava, inferior vena cava and coronary sinus were present and unusually connected to the left atrium. Surgical correction of the anomaly was successfully achieved [35]. Overall, experts agree that if the PLSVC, which drains into the left atrium, leads to a large right-to-left shunt, the patients should undergo surgical correction [35,36].

In the context of device implantation, the clinical importance of PLSVC aberrant drainage into the left atrium is major, especially in the situation of abnormal anatomy of the right superior vena cava, which creates an unfeasible anatomical scenario for endovascular pacemaker lead placement. Acknowledging such anomalies and anatomical variants is essential, especially in the field of electrophysiology, in order to avoid vascular complications and to make sure the appropriate technique is used for patients requiring cardiac devices. Considering the challenges of these cases, a multidisciplinary team consisting of radiologists, sonographers, electrophysiologists, anesthesiologists and cardiothoracic surgeons should collaborate in terms of surgical correction, cardiac device placement, etc., in order to avoid possible complications.

## 4. Conclusions

We reported the case of a patient with significant conduction disorder associating a complex thoracic venous anomaly that required an interdisciplinary team for successful pacemaker implantation. Although rare and usually asymptomatic, such abnormalities are technically challenging; thus, it is of major importance for the electrophysiologist to be familiar with thorax venous anomalies and the different strategies for cardiac device implantation. Since many of these venous abnormalities may coexist with other congenital disorders, a careful stepwise evaluation using different imaging techniques is mandatory for proper management.
